# Rectal treatment in ulcerative colitis; a qualitative study exploring reasons for under utilisation

**DOI:** 10.3389/frhs.2025.1654520

**Published:** 2025-10-01

**Authors:** Jean-Pierre Rothen, Florence Moerlen, Céline von Büren, Samuel Allemann, Isabelle Arnet

**Affiliations:** Pharmaceutical Care Research Group, Department of Pharmaceutical Sciences, University of Basel, Basel, Switzerland

**Keywords:** rectal treatment, ulcerative colitis, adherence, real-world data, stakeholder

## Abstract

**Introduction:**

Ulcerative colitis is an inflammatory bowel disease that is limited to the colon. First-line treatment consists of rectally administered suppositories, foam or enema. Adherence to rectal treatment is very low in people with ulcerative colitis. We aimed to explore modifiable factors influencing adherence to rectal treatment among patients, healthcare providers (HCP), and pharmaceutical companies, and to delineate new approaches to improve adherence to rectal medicines.

**Methods:**

We recruited people using rectal treatment, community and clinical pharmacists, gastroenterologists, inflammatory bowel diseases (IBD) nurses, and representatives of pharmaceutical companies specialised in the production of rectal therapies. We performed semi-structured one-to-one interviews tackling the three pivotal topics knowledge and beliefs, product characteristics, and communication. We started with patient interviews whose statements served to inform the other interviews. All interviews were the subject of a keyword protocol, audio-recorded, and transcribed. Thematic analysis was used with inductive approach for the patients, and deductive approach for all other interviewees. Identified themes were compared and agreements and divergences were compiled.

**Results:**

We interviewed eight patients (22–77 years old, 3 women) in spring 2023, and stakeholders from the hospital (3 gastroenterologists and 2 IBD nurses), ambulatory setting (4 community pharmacists) and pharmaceutical companies (2 representatives) in 2024, all in the region of Basel (Switzerland). Overall, people with ulcerative colitis often feel left alone to cope with the challenges associated with their therapies. Pretreatment concerns and difficulties experienced by these people following their first attempts at use are not adequately addressed by HCPs. Training sessions for clinicians and pharmacists, patient-oriented demonstration materials, visual aids, and tips and tricks the application of rectal treatment could help improve the use of rectal medicines by people with ulcerative colitis.

**Conclusion:**

This study identified a detailed knowledge of the proper use of rectal treatment and frank communication between patients and HCPs as crucial for the adherence to rectally administered medicines. While these factors are broadly recognised in the literature, this study highlights their common priority among all stakeholders. In addition, solutions for future development and tailored interventions are proposed.

## Introduction

Ulcerative colitis is a chronic relapsing inflammatory bowel disease (IBD) that is limited to the colon. Some subgroups exist by extent of disease, that are: ulcerative proctitis (involvement limited to the rectum), left-sided ulcerative colitis (limited to the portion of the colon distal to the splenic flexure), and pancolitis (extended proximal to the splenic flexure) (1).

According to ECCO (European Crohn's and Colitis Organisation) guidelines (2, 3), 5-aminosalicylates (5-ASA) are the established treatment for ulcerative colitis. 5-ASA is often administered orally only, although 5-ASA products are more effective when administered both rectally and orally, particularly in proctitis and left-sided colitis (4). Alternatively, corticosteroids may be used, which can be administered rectally, orally or intravenously. The subgroup pancolitis requires a combination of rectal and oral treatment (2). In addition, immunosuppressants and biologics are increasingly used, and are often initiated with top-down rather than a step-up approach (5). These treatments are significantly more expensive than rectal therapies. In contrast, low-cost alternative treatments often refer to therapies used alongside conventional medical approaches such as dietary or nutritional approaches, and mind-body therapies.

**Figure 1:**
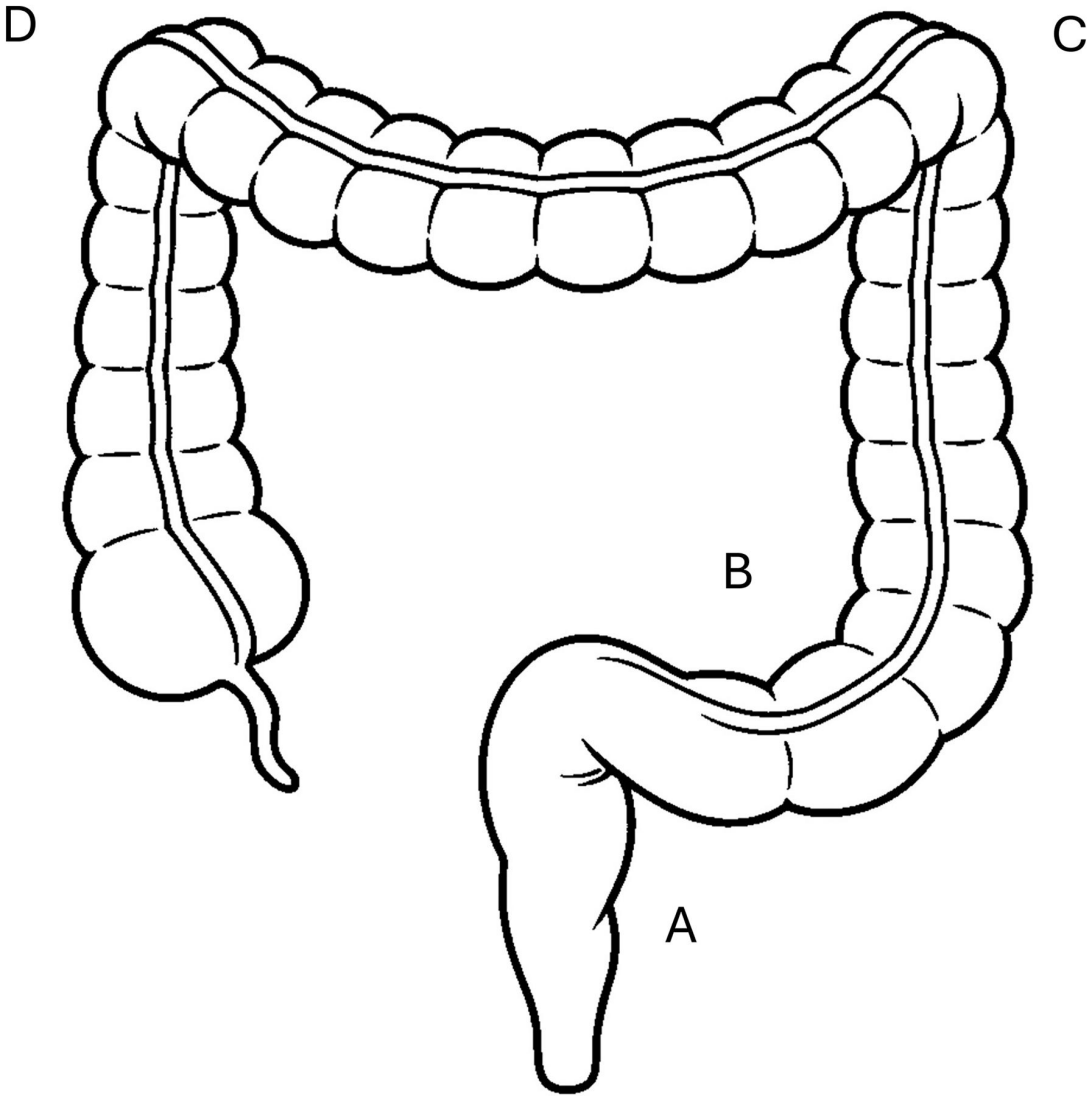
Most inflammations with ulcerative colitis are limited to the rectum **(A)** or left side **(C)** of the colon, while pancolitis is less common. Effects of suppositories are limited to the rectum **(A)**, rectal foams reach the sigmoid **(B)** and descending colon **(C)**, enemas can reach up to the splenic flexure **(D)**.

First-line treatment occurs rectally with suppositories, foam or enemas (Figure 1), and is particularly effective when the inflammation affects only the distal region of the colon (4, 6). Because rectally administered medicines reach the site of inflammation quickly, rectal treatment is more effective than oral treatment and is often associated with fewer adverse events. However, the active ingredient must be kept in the rectum at the site of application for 20–30 min for an optimal effect (7). Clinical studies have shown that adherence to rectal treatment in ulcerative colitis is very low. In a cohort of 70 patients with ulcerative colitis, 71% were non-adherent to rectal 5-ASA (8). Similarly, in a study with 485 IBD patients answering a questionnaire, oral treatment was associated with a significantly higher adherence (60%) than rectal treatment (32%) (9).

Poor adherence to IBD treatment is associated with increased disease activity and flare-ups, reduced effectiveness of medication, diminished quality of life, and a heightened risk of colorectal cancer (10). In a study including 99 ulcerative colitis patients in remission, adherent patients had an 89% chance of maintaining remission compared with 39% in those who were non-adherent (11).

Next to the disease burden and associated complications for patients, families and carers, costs represent a further challenge. Direct costs of care for people with IBD are more than 3-fold higher compared with non-IBD, and are principally due to medicines, comorbidities and emergency department use (12). The indirect costs relate to restrictions in daily life, time spent on health care and absence from work (12). In a study with more than 4,000 colitis patients with prescription of 5-ASA, gastroenterology-related inpatient costs were twice as high for non-adherent patients compared to adherent patients (13).

Important factors for non-adherence in people with ulcerative colitis have been identified, including patient-related such as younger age, single status, male gender, full-time employment, lack of belief in the effectiveness of medication, fear of side effects; and disease-related such as symptomatic remission, recent diagnosis; or medicine-related such as complexity of administration regimen, invasiveness of the rectal formulation, concomitant medications (10, 14). However, only few factors are modifiable and thus, are likely to change following an appropriate intervention. We extracted modifiable reasons for non-adherence from literature (15, 16) and selected those most relevant to rectally applied medicines.

The problem of poor adherence to rectal treatment is well known, including among pharmaceutical companies. They are considering this during product development and contribute perspectives on modifiable factors rooted in their core expertise. For example, one option for improvement is to ameliorate the application such as a temperature-triggered *in situ* forming lipid gel (17). We did, however, not focus on specific product development due to the rapid change in this area.

We aimed to explore modifiable factors of non-adherence to rectally applied medicines from the perspectives of concerned people, gastroenterologists, IBD nurses, clinical pharmacists, community pharmacists, and pharmaceutical companies specialised in the production of rectal medicine. Finally, we identified approaches to improve adherence to rectal treatment.

## Methods

### Research design

An explorative design was selected with one-to-one semi-structured interviews conducted in the region of Basel. Interviews were conducted at locations chosen by participants, including seminar rooms at the University of Basel or in community pharmacies, hospital waiting rooms, or the people's home.

### Sampling and sample

Inclusion criteria for patients were people aged 18 years and older with ulcerative colitis for at least 18 months and an individual experience in the use of rectal treatment. Recruitment was performed through flyers advertising the study that were handed out to people entering the Clarunis (University Digestive Health Care Center) at its two sites, St. Clara Hospital and University Hospital Basel. Participants were invited to share their personal experiences with rectally administered medicines with researchers from the Pharmaceutical Care Research Group at the University of Basel, Switzerland. Interested persons could contact the researcher (JR) by e-mail or telephone and fix an appointment. The remaining stakeholders were recruited via personal contacts by e-mail or telephone. Two interviews were conducted via Zoom. Written consent was obtained before the interview.

### Interview guide for concerned people

Modifiable factors of non-adherence to rectally applied medicines in IBD-patients were screened in literature and reviewed against personal experience by the authors. Tackling knowledge and beliefs (such as a lack of understanding of the disease and of the drug regimen, fear of side effects, the medication itself (such as inconvenient or complex dosing), product characteristics, and communication (such as shortage of information, lack of belief in HCP's judgement (10, 18) were judged relevant. The interview guide consisted of 3 blocs and nine open-ended questions (see Appendix 1). Each bloc addressed one factor of non-adherence. The interview guide was developed and tested for comprehensibility, first with the investigators (JR, IA) and second with a person with IBD not involved in the study (man, 30 years). Each bloc started with a closed-end question that introduces the topic of the bloc. The interview started and ended with a mood question: “How do you feel at the moment?” with an answer scale from 1 (very uncomfortable) to 10 (absolutely fine). To allow people to report their personal experiences in their own language, the scientific term adherence was not used directly.

The first bloc addressed on knowledge and inquires how long people have been living with ulcerative colitis, and their individual experiences with the rectal application of medicines. Knowledge about the benefits of the rectal administration of medicine was asked indirectly through the degree of conviction that rectal application leads to successful treatment with an answer option between 1 (not at all convinced) and 10 (totally convinced).

The second bloc addressed medication by asking for the optimal product, the optimal patient information leaflet, and the analysis of the patient information leaflets of commercially available mesalazine and budesonide. By focusing on the best product, we suppose that participants referred mentally to their negative experiences and prioritize them before formulating their answers. This saved time.

The third bloc addressed communication and enquired about the first reaction after having been prescribed a rectal product, after receipt of the product at the pharmacy, about the explanations received from HCPs, and concerns and eventual difficulties reported to the HCPs.

The researchers (JR, IA) jointly reviewed the questions to ensure they were understandable.

### Mirroring people's statements, and interviewing community and clinical pharmacists, gastroenterologists, IBD nurses, and pharmaceutical companies' representatives

The findings from the patient statements were mirrored to the other stakeholders where applicable (see Appendix 2). As an example, the person answers to the question on their knowledge about the benefits of rectal treatment were transformed into “*In which situations do you recommend rectal treatment?*” for prescribers and “*what benefit do you mention?*” for prescribers and for pharmacists.

The guide for other stakeholders was structured similarly to the patient interview guide: (1) Main reasons for rectal treatment and personal practice (2) Optimal product and (3) Communication with patients. The stakeholders were invited to comment on patients' statements and reveal their personal views. Two answer options were dichotomous (yes/no) with a supplementary question on the reasons for the answer given.

The starting questions of patient's knowledge was raised with the other stakeholders by asking when rectal treatment is used, and what benefits are pointed out.

Secondly, patients' responses to the optimal rectal product were reflected. Further, there was an assessment of the future market opportunities of a new type of galenic formulation (17).

Regarding communication, HCPs were asked how they motivate the patients for rectal treatment; what explanations they give; how they deal with patient's concerns before treatment and difficulties after the first attempts, and what might be the reasons why not all patients seek professional help despite their need. Pharmaceutical companies' representatives were asked whether specific concerns of the HCPs reached them; whether they could meet the wishes of pharmacists for training on the products; the wish expressed by a gastroenterologist for training devices or the wish expressed by an IBD nurse for products with smaller volumes.

Finally, all interviewees were asked if they wanted to share anything else about rectal treatment.

### Data collection

All interviews were conducted in Swiss German by one researcher (JR) but one in English for one pharmaceutical company representative. With patients, the interviewer took simultaneously field notes of the main statements. With the remaining stakeholders, another researcher (FM) took field notes of the main statements. The interviews were audio-recorded.

### Data analysis

Averages with ranges were calculated for the answers to the Likert scales.

Within the blocs, we used thematic analysis according to Braun (19) to analyse the interviews of the patients and the remaining stakeholders separately. This method is more interpretative and involves insight into participants' experiences. It identifies main themes rather than categories and provides depth to the analysis. The audio recordings were used by a second researcher (CvB for patients; FM for the remaining stakeholders) to complement the field notes of the main statements. Initial themes were generated by the researchers (CvB and FM) and JR separately to ensure rigor. After iterative discussion and consensus, seven themes were identified from the patient's interviews. Relevant quotes were forward translated into English by an investigator (JR).

### Suggestions for the improvement of the adherence to the rectal treatment of ulcerative colitis

Mirrored answers and patient's statements were compared. We extrapolated potential improvement from agreements and divergences.

### Ethical considerations

We obtained approval from the local ethical committee (EKNZ 2022-00899, SNCTP 000005074). The study is reported according to the COREQ reporting checklist.

## Results

People concerned were recruited between February and April 2023 and one-to-one meetings were conducted. Nine accepted the participation, one without any experience of rectal treatment was excluded. The remaining eight people (22–77 years old, three women) were interviewed in March and April 2023. They were living with ulcerative colitis for 5–21 years and had individual experience with the use of rectal formulations.

A total of 12 HCPs were invited. Three gastroenterologists refused participation and no clinical pharmacist familiar with ulcerative colitis and rectal dosage forms could be found in the three hospitals in the region of Basel. A total of nine interviews were conducted in March and April 2024 with three gastroenterologists and two IBD nurses working in hospitals, four community pharmacists in Basel City and two representatives of pharmaceutical companies.

### Answers to mood expectations and willingness

The people's mood was on average 7.6 (range: 3–10) at the begin of the interview, and 8.5 (range: 5–10) at the end of the interview. Conviction about the benefits of the rectal administration of colitis medicines was 7.5 (range: 3–10). Expectation of a detailed explanation at the pharmacy was 1.5 (range: 1–2) and willingness to continue after the first experience was 7.0 (range: 3–10).

### Main themes

Seven main themes emerged from the patients' interviews that are presented here with the corresponding views of the remaining stakeholders:

1. Awareness of the advantages of rectal treatment

All people concerned with ulcerative colitis were able to describe a benefit of the rectal treatment. Sometimes HCPs pointed out the benefits of rectal treatment. Some community pharmacists only encourage their patients when asked. All gastroenterologists recommend rectal treatment for proctitis up to left side colitis, but not for pancolitis. They consider rectal treatment a good and often successful form of treatment with few adverse events and economical aspects. In addition, people with IBD can intensify the treatment if necessary and thus, get a sense of control over the disease. In recent years, some gastroenterologists are increasingly treating according to the top-down principle and start the treatment with the prescription of biologics. One gastroenterologist selects the treatment explicitly according to the shared decision-making principle.

IBD nurses recommend rectal formulations particularly in acute relapses, also as a supplement to biologics. They point out that it is an evidence-based method. Depending on the extent of the inflammation and the living conditions of the people concerned, the most suitable product is chosen. The prescription is made in cooperation with the responsible gastroenterologists.

2. Concerns about the prescribed medicine

Many people with IBD have concerns when a rectal treatment is suggested, what they express as follows: “Does it have to be? Something to swallow would be easier” (male, 77 years); “How am I supposed to keep this inside?” (male, 42 years). Sometimes patients address their concerns to the prescribers but they don't always dare. “You don't know what's coming; it's not a nice moment” (male, 52 years). Rectal treatment concerns the anal region and thus, people with IBD have difficulty raising concerns to someone they do not know. Therefore, sometimes they do not dare to ask.

When it comes to people's privacy, general recommendations are of little help. Gastroenterologists give general instructions with the initial prescription, especially for rectal foam and enemas, and less so for suppositories. Two gastroenterologists proactively address potential difficulties of a rectal medicine during the consultation; one refrains from any explanation to not frighten the people with IBD. If some people with IBD are worried, the IBD nurses share stories of similar mishaps experienced by others to help them feel less alone in their difficulties. Pharmacists mentioned that people with IBD hardly raise their concerns in the community pharmacy. The community pharmacists show good will and provide information when requested, but rarely address potential concerns in a proactive way. This is due to time pressure, but also to a lack of knowledge regarding the diagnosis and insufficient knowledge regarding how to carry out rectal treatments, as admitted self-critically (female, 49 years).

3. Reporting application difficulties

People with IBD commonly report difficulties including bowel urgency, burning, pain during insertion and application. Rectal treatment can be painful in an acute inflammation, and each stretch of the inflamed colon causes further pain (IBD nurse). The larger the volume applied, the greater the difficulties. Sometimes, rectal treatment is just not possible due to the strong rectal inflammation.

All experience rectal tenesmus after application as a major problem. Some were surprised to learn during the interviews that they were not the only ones with bowel urgency (male, 42 years). When people with IBD report struggling, doctors sometimes respond with recommendations that are not very helpful such as, “Didn't it work? Try again!” (male, 41 years; male, 27 years). One (male, 27 years) described his sense of shame when he was first prescribed rectal treatment at the age of 15 and had to ask his mother for help. He particularly would have needed empathetic professional support. Other difficulties relate to hygiene. One (female, 66 years) reported that her gastroenterologist had explained that a small amount of the introduced fluid could leak out. She found it helpful to know that this was normal and that it could be handled with a towel as a bedding.

All gastroenterologists ask patients at the follow-up consultation how they have coped with rectal treatment. IBD nurses proactively address application difficulties. People with IBD can contact them by phone or email. Pharmacists find again that people with IBD do not dare to report their application difficulties in the pharmacy. Pharmacists imagine that it is due to lack of time or if they do not feel taken seriously enough, people with IBD often refrain from further inquiries. Reports from HCPs reach the medical team of the pharmaceutical companies via sales representatives. Sometimes people with IBD report directly, which is appreciated.

4. Motivation for rectal treatment

In spite of concerns, people with IBD tend not to be fundamentally negative towards rectal treatment. One (male, 41 years) reported, “over time, I preferred rectal to oral cortisone; it works faster and a lower dose is needed”.

IBD nurses consider the motivation of people with IBD as their main task. Sometimes, they use studies to argue particularly with people with IBD who respond well to evidence-based arguments (female 59 years). Not all people with IBD have been allocated an IBD nurse. For gastroenterologists, motivation is associated with the psychological stress of the people with IBD. Two of three gastroenterologists choose a product together with the patients. Various circumstances prevent from motivational support in pharmacies: time pressure; ensuring discretion (use the consultation room or not?). Pharmacists usually assume that the people with IBD have been given sufficiently information about the treatment. Many pharmacists recognise a lack of practical knowledge and giving incomplete instructions to patients.

5. Visual information (pictures or brochures)

Pictures are important for patient information, especially for those who have received inadequate instructions from the HCPs. The picture of a standing patient during a rectal application in a package leaflet is misleading (male, 41 years). A read-friendly structure of the text is also crucial.

All gastroenterologists use pictures, sometimes even sketched by themselves that people with IBD are allowed to take with them. IBD nurses work with pictures and texts. Visualisation is particularly valuable for people with IBD with reading or language difficulties. Visual copies of the devices for the instruction could be helpful (4 patients). This view is confirmed by all other stakeholders. Instruction with a mock device while prescribing a medication could be a good training. However, demo devices are no longer provided by companies (gastroenterologist, 73 years). Some training opportunities from pharmaceutical companies for HCPs exist, but the availability is not widely known.

Pharmacists think that there is too little information available. The representatives of pharmaceutical companies offer brochures for physicians but have rarely direct contact with the pharmacies. Ultimately, pharmaceutical companies leave it to the physicians to decide on how to use the information material (both representatives of pharmaceutical companies).

6. QR code on patient material

A QR code on the package or the patient information leaflets with a link to an application-video is approved by all people with IBD.

When asked about QR codes, pharmacists consider it a good idea for the future. An IBD nurse expressed concerns about data protection because data could be traced by health insurance companies. One representative of pharmaceutical company has already realised a QR code in a brochure. However, a QR code would be difficult to realise on officially approved material.

7. Tips and tricks

Tips and tricks which should be shared with the patients were reported by all stakeholder and include the following: Rectal formulations are best applied lying down, best on the left side. For enema and rectal foam, a towel should be placed underneath the bottom; it is normal for a small amount of liquid to leak out. Most people with IBD experience less discomfort when the product is warmed before application. The pump head of the rectal foam should be pushed, hold in this position for about 2 s and then released very slowly. Ointment, lubricant, if necessary, also local anaesthetics can facilitate the introduction of the device. For some people, an insert in the underpants may be useful. People with IBD should also be aware that correct application requires patience. With practice, there is a good chance that later attempts will be more successful.

### Suggestions for adherence improvement to rectal treatment of ulcerative colitis

People with IBD mentioned some practical suggestions for improving adherence in rectal treatment of ulcerative colitis: The addition of a local anaesthetic to make insertion less painful; pre-greasing of the enema to facilitate insertion; a conical blunt attachment for rectal foam and enema with openings on the side instead of a cylindrical stem with corners that can cause pain in the inflamed rectum. With a spray catheter, which merely wet the mucous membrane, a smaller volume would be introduced, which would reduce the urgent feeling to pass stool.

Further suggestions can be found in the HCP's responses (Table 1). Ideal rectal medicines with an optimal formulation should not cause bowel urgency; the packaging should enable to take the product with you; rectal foam is impractical due to the large packaging. In addition, unit-item packaging produces a lot of waste. It is important that patients dare to address their concerns and difficulties to any HCP involved. Consistent counselling from different HCPs would make it easier to deal with patients' worries.

**Table 1:** Difficulties reported and claims for measures to improve adherence to rectal treatment in ulcerative colitis patients.

Stakeholder	Difficulty	Claim
Patients	Timidity	Address personal concerns and difficulties.
Gastroenterologists	Compliance with the guidelines, lack of time	Follow the guidelines, and spend as much time as possible with patients.
IBD Nurses	Scarce number	Be hired in greater numbers in hospitals.
Clinical pharmacists	Involvement in patient's care	Play their intended role in the counselling of patients.
Community pharmacists	Involvement in patient's care	Have more knowledge about IBD and rectal treatment.
Pharmaceutical companies	Support of HCPs	Offer trainings and provide information material to all HCPs and training devices.

Quality circles and professional associations could invite the pharmaceutical companies to present the proper handling of their devices. Their representatives are positive about this interest.

## Discussion

Interviews about rectal treatment with people with colitis enabled to identify themes that hinder successful use of rectal treatment. The statements were then mirrored by HCPs and pharmaceutical companies' representatives.

### Stakeholder views

All stakeholders were aware of the benefits of a rectal treatment, which is a key component in adherence. It is well-known that the concerns of people with IBD about treatment efficacy negatively influence medication adherence (20). Consequently, concerns and application difficulties should be actively addressed by HCPs. However, in-depth conversations with people with IBD would require more time than available. Clinical pharmacists are not involved in the process in the region studied. Their task is to evaluate the appropriateness and effectiveness of the patient's medications. Although community pharmacies dispense rectal medicines, they are rather passive and play a minor role in counselling. Peoples’ expectations of pharmacy advice are very low. Training for pharmacists on IBD and rectal treatments could encourage more in-depth counselling.

### Stakeholder solutions

It is important that the people with IBD feel personally addressed and have the opportunity to read information at home if necessary. Accordingly, in a study with colitis patients, tailor-made counselling in the intervention group improved adherence by 44% compared to a control group (21). For prescribers, biologics prompt the option of a simple, successful, but expensive treatment (5) which additionally does not fully comply with the recommendations of the guidelines.

The proposed solutions should lead to an optimization of rectal products, from the developer to the enduser. Practical barriers to their implementation might include cost, regulatory constraints, and resource availability. However, training for pharmacists on IBD and rectal treatments could encourage more in-depth counselling with manageable efforts.

### Adherence

Adherence is an abstract term, mostly unknown to patients (20). In addition, when used among HCPs, it may evoke some personal entities close to conditioning. For this reason, the terms adherence or ancestral compliance were avoided throughout the interviews. One of the advantages is that interviewees could speak freely without being primed by the term. One of the risks is the ambiguity of the wording, which can lead to unexpected answers and statements. However, this is a disadvantage of any interview.

### Generalisability

The degree of cooperation of the different stakeholders might differ from one country to another. As an example, clinical pharmacists in other regions may be more involved in the care of colitis patients. Thus, regional setting may limit the generalisability of our investigation. Nevertheless, the new aspects delivered by our study might be useful for all healthcare providers and developers of rectal formulations.

### Strengths and limitations

Our study has some strengths. First, we targeted all stakeholders involved in the rectal treatment of ulcerative colitis: people with IBD concerned, and also gastroenterologists, IBD nurses, community pharmacists, and pharmaceutical companies' representatives. Although we were unable to gain clinical pharmacists working in an hospital, we claim that the proposed actions are conclusive.

Second, we concentrated our interviews on modifiable determinants and practical measures. Compared to other studies that identified unmodifiable factors of non-adherence such as age, single status, male gender or full-time employment (10), we claim that our results can be used to develop practical measures to ameliorate the use of rectal medicines.

Third, interviews were conducted until data saturation. It is likely that recruiting more individuals might have allowed minor additional insights.

We acknowledge some limitations. First, the established roles of the different stakeholders found in our setting are not entirely transferable to other countries. Nevertheless, our suggestions such as the improvement of communication (18) and interprofessional cooperation are generally valid.

Second, the interviews were conducted with a limited number of stakeholders. It is possible that not all aspects were mentioned. The interviews were not transcribed verbatim, which could mean a loss of information. However, the combination of written notes and audio recordings made it possible to identify concise statements. Altogether, we claim that the main statements were mentioned and that the extrapolated themes are representative.

## Conclusion

In conclusion, we were able to explore reasons for poor adherence to rectally used medicines in people living with ulcerative colitis. Adherence to rectal treatment could be improved with visual explanations that may be easier to understand than complicated texts. Moreover, HCPs should actively address their patients' concerns and difficulties and provide tailored information and training with mock devices. Increased knowledge of the disease and a better understanding of treatment could potentially help people with IBD to improve their adherence to rectally administered medicines.

## Data Availability

The raw data supporting the conclusions of this article will be made available by the authors, without undue reservation.

## Appendix

Appendix 1 Interview guide for patients.

**Table:**

Topic	Item
Introduction	Please express how you feel at the moment. Choose a number from 1 (very uncomfortable) to 10 (perfectly fine).
Knowledge and personal experience	How long you have been living with ulcerative colitis?
	Do you already have experience with the rectal application of medication?
	What is the name of the product? How often do you use the product?
	Do you know the benefits of rectal use of medication for your condition? How would you describe these benefits in your own words?
	If you as a patient apply active substances directly, i.e., rectally in the colon, you contribute to the success of the treatment. How convinced are you that rectal application leads to successful treatment? Select a number between 1 (not at all convinced) and 10 (convinced)?
	Now I would like to know in more detail which rectal products you have already used yourself: Suppositories, rectal foam, enemas or other galenic formulations? What was your experience with these products?
Optimal product	Let us now turn to the optimal product, starting with the product itself and its application. What would characterise an optimal rectal product?
	What characteristics should an optimal rectal foam have?
	Let us now turn on to the optimal information about the product. What should the optimal information about the product look like?
	Would, for example, a QR code with a link to a film on how to use the product correctly be useful?
	Here we have three examples of patient information on rectal medication for ulcerative colitis: What do you think is good/what do you think is less good?
Communication	Now let's turn to the person who is supposed to give you the best possible explanations about the product. Remember the moment when you were prescribed a rectal product for the first time by your doctor. What were your initial reactions in the doctor's office/hospital?
	Remember the moment when you were given a rectal product for the first time in the pharmacy: What were your initial reactions?
	You were given explanations, either by the doctor or in the pharmacy. Was this explanation to your satisfaction? What was good/what was less good?
	Would you have been glad to receive a detailed explanation from the pharmacy on how to use the product? Select a number between 1 (no, not necessary) and 10 (yes, absolutely necessary).
	Many people have concerns about rectal application. Do you also have concerns and if so, what are they?
	Have you reported your concerns to your doctor or pharmacy? If no: What prevented you from expressing your concerns?
	And then you used the rectal product for the first time. How willing were you to use the product a second time? Choose a number between 1 (not at all) and 10 (absolutely).
	You have previously indicated with the number XX how convinced you are of the effect of rectal application. What would it take for you to be even more convinced of the success of this treatment?
Final questions	Is there anything else you would like to tell us about rectal treatment?
	And now please express how you feel after the interview. Choose a number between 1 (very uncomfortable) and 10 (perfectly fine).

Appendix 2 Interview guide for remaining stakeholders.

**Table:**

Topic	Gastroenterologists, IBD nurses	Pharmacists	Pharmaceutical companies
Personal practice	In which situations do you recommend rectal treatment? [question only for gastroenterologists]		
	In which situations do you not suggest rectal treatment? [question only for gastroenterologists]		
	When counselling a patient about rectal treatment, what benefits do you mention?	When counselling a patient, what benefits of rectal treatment do you mention?	What are the main reasons for a rectal treatment?
		When do you recommend a specific formulation?	
Optimal product	What criteria do you use to make a recommendation for a product (e.g., for the galenic form)?		What criteria do you use to decide on the development of a particular galenic form?
			Last year's publication describes a formulation that presents itself as a gel at room temperature. In the rectum at body temperature, it changes its structure, becomes viscous and lays down like a film on the mucous membrane. This requires a much smaller volume, which, as we know, triggers the urge to stool in many patients. Are you familiar with the study?
	Who decides which product to choose (gastroenterologist, patient, or both together)?		
			HCPs prefer neutral information. But as a company, you want to position yourself. How do you deal with the different interests?
			Are your brochures available to patients? If yes: What kind of illustration items are available? How do you bring illustrative material into the consultation? If no: Why not?
	In the interviews, patients were asked what they thought the best information about a rectal treatment should look like. Most patients thought pictures and text were important. Do you use pictures, visual materials, etc.? If yes: what tools do you use? If no, why not?		In the interviews, patients were asked what the best information on a rectal preparation should look like for them. Most patients felt that pictures and text were important. Do you have any brochures, pictures or other illustrative materials available to professionals? If no: Why not?
	All of the patients interviewed were in favour of a QR code that would allow them to look up all the important information on their mobile phone and find instructions, pictures or videos on how to use them. What is your opinion?		
			If you approve of the idea with the QR code: Could you imagine printing it on the package leaflet or on package box?
Communication	How do motivate the patients for a rectal treatment? Many patients lack empathy because rectal treatment may cause difficulties. How do you explain when you first prescribe/dispense a rectal treatment? Many patients need counselling, for example if the application of a rectal treatment does not work right away.		
	What problems do you raise?		
	Do patients contact you if they have difficulties with rectal treatment? If yes: Do you have examples of problems that have been brought to your attention? If no: Do you deliberately ask your patients at the next dispensing how it worked?		Do HCPs contact you when their patients have difficulties with rectal treatment?
		How do you grant discretion in the pharmacy if further instructions are needed?	
	The majority of interviewees had initial concerns about rectal treatment, but only about half of them reported these concerns to their doctor or pharmacist. Do you have any idea why not all patients dare to report their concerns to a HCP?		
			Some pharmacists told us that rectal treatment is not subject to continuing training courses. Could you imagine as a company providing training for professionals?
			A gastroenterologist reported of a training device for a rectal foam, which had been available in the past, but now no longer. What could be the reason?
			An IBD nurse reported that, especially in acute relapses, the elongation of the inflamed colon causes so much pain for many that it is impossible for them to apply large-volume preparations. So, she wants smaller-volume preparations. What is your advice?
			A gastroenterologist told us about tacrolimus suppositories for persistent proctitis. These are produced by pharmacies in recipes. Do you see any potential for such a product?
Final question	Would you like to share with us anything else about rectal treatment?		
